# Estimation of Psychological Stress in Humans: A Combination of Theory and Practice

**DOI:** 10.1371/journal.pone.0063044

**Published:** 2013-05-15

**Authors:** Parul Sood, Sushri Priyadarshini, Palok Aich

**Affiliations:** School of Biological Sciences, National Institute of Science Education and Research (NISER), Institute of Physics Campus, Sachivalaya Marg, Odisha, India; Semmelweis University, Hungary

## Abstract

Stress has long been known to increase susceptibility to health disorders. In 2009, American Psychological Association further established association of stress to serious health problems. However, a quantitative and accurate way to evaluate and estimate stress status of individuals is still a big challenge. It has been shown, in large animal models using cattle, that psychological stress can be quantified as well as disease susceptibility could be predicted through biomarker discovery. Taking cue from those studies, we have evaluated and estimated psychological stress level of individuals theoretically and validated experimentally. Various biomarkers have also been identified which can be associated to psychological stress to predict stress status of unknown individuals.

## Introduction

Modern day life usually demands more work than relaxation. Consequence of such situation may be grim in terms of health. According to a report by the American Psychological Association, 33% of Americans are living with extreme stress and 48% of Americans believe that their stress has increased over the past five years [1]. More studies are required to establish these facts globally. In general, our perception is that individuals in any part of the world are experiencing more stress in daily life than before, leading to disease susceptibility. Stress is affecting both physically and socially. It is thought for long that consequence of stress may lead to health problems, poor relationships and loss of productivity at work. In 2009, it was further related to serious health related problems [2], [3]. It has been shown recently in cattle model that psychological stress (PS) has huge impact on etiology and outcome of bovine respiratory disease (BRD) as well as biomarkers were associated with PS to predict BRD outcome [4], [5].

Stress, in general terms, is defined as any kind of disturbance in physiological homeostasis [6]. Thus, PS is the homeostatic alteration caused by psychological factors which may include various social and emotional stressors. The concept of PS has slowly evolved from a neurobiological to a neuro-physiological basis. This development is evident from the methods of qualitative and quantitative assessment of stress, which have improvised from the classical questionnaire based evaluation of stress to current molecular screening methods. Initially, PS was considered purely from a psychiatry viewpoint. As a consequence, various questionnaires were used and are still used for the psychological assessment of an individual. These questionnaires are designed based on various factors like daily hassles, happiness scale, perception of the present and the future by an individual (e.g. optimism or pessimism), personality traits, depressive life events, and so on [7], [8], [9], [10]. Although these questionnaires, with certain scoring methods, can assess the degree of stress involved, but there are several drawbacks, such as, a) the subjective bias of an individual towards the assessment of his/her psychological state, b) the tendency of an individual to maintain secrecy regarding personal matters while avoiding any disclosure even when confidentiality is maintained, c) the limitation of questionnaires in terms of their objectivity thus restricted to investigate an individual without prior bias. All these factors, therefore, pose an upper limit to questionnaire based evaluation of an individual for PS and raise serious queries regarding validation of the estimation. Thus, for a more comprehensive and robust evaluation of PS, hormonal assays came into picture in which levels of epinephrine, norepinephrine, and cortisol are checked to differentiate various stressed conditions [11]. In addition, other physiological studies which include estimation of various clinical parameters, such as elevated blood pressure, rise in body temperature and changes in body weight, have also been used to measure PS response [12]. However, these studies failed to estimate and quantify degree of PS in different scenarios [13].

These problems, associated with hormonal assays and physiological readings, have been dealt with by expanding the studies of PS quantification to further downstream products of stress response rather than restricting to primary hormonal responses as well as using various tissues to acquire the final read-outs. As a result, recent studies have shown the differentiation of stressed patients based on various pro-inflammatory cytokines, acute phase proteins and minerals which are supposed to be activated in response to a stimulus of PS [14], [15], [16]. However, these studies treated physiological processes such as immunity or neuro-signaling or metabolism independently and the effects of stressors, on these processes, were studied in isolation.

It is, therefore, required to take a holistic approach to stress which serves in two ways; 1) provides a consistent, reliable and effective biomarker level and duration for stress quantification, 2) gives an idea of the molecular basis of PS in relation to modulation of physiological homeostasis. We have characterized metabolite and proteomic profiles and integrated with modified classical questionnaire approach to address the issues of PS [17]. In the current study, our approach is not to evaluate stress status by constituting experimental and artificial stress situation for a group of individuals rather we try to evaluate the natural stress status of individuals as a result of dealing with regular chores and to see if methodologies we employed are sensitive enough to evaluate and understand the biology of stress status and condition. We hypothesize, that a combination of theoretical and experimental molecular approaches would be able to distinguish individuals in terms of stress condition and level.

### Ethics Statement

Written consent from all individuals for their voluntary participation was obtained before beginning of the study as per the ethical requirement to conduct study and the study was approved by the Human Ethics Committee, National Institute of Science Education and Research (NISER). There were no minors/children participants in the study.

## Materials and Methods

### Subjects

A cohort of random local population was selected for the current study. Selected individuals were otherwise healthy and belong to a group who maintains normal lifestyle, mostly do not smoke and drink alcohol. Both males and females were included in the study whose age ranged from 18–55 yrs with weight ranging from 45 kg to 85 kg (mean weight 65[±3] kg, height ranging between 140 cm and 185 cm [mean height 168(±4) cm] and BMI ranged between 21 and 26. Approximately 4 ml of blood was withdrawn from each subject by a professional and trained nurse from local hospitals or clinic using standard operating procedure (SOP). Blood was collected from 135 individuals and stored in SST tubes for serum processing as mentioned elsewhere.

### Questionnaire

The questionnaire used for preliminary quantification of stress for individuals in the population was divided into three parts; Part A, Part B and Part C (Questionnaire S1). Questionnaire is available online at http://oa.niser.ac.in/SMS/login.htm.

Part A was a stress questionnaire that identified people as stressed (S), non-stressed(NS) and borderline (BL) depending on first standard deviation interval of the distribution plots acquired from the total questionnaire scores (Q-scores) of each individual. This part took into consideration two aspects associated with psychological stress; a) Physiological, b) Psychological. The questionnaire thus included questions based on the symptoms associated with stress like excessive sweating, severe headaches, insomnia, fatigue and psychological factors like absent-mindedness, procrastination, jealousy [7], [8], [9], [10].

Part B dealt with grouping people into chronically stressed or acute stressed. For this classification, two standard questionnaires were used; a) Holmes and Rahe stress scale [18], [19], [20] which scored the major life change units and thus gives an indication of risk of illness, mental or physical faced by a person based on stress, b) Hari's Stress Inventory [21] which accounted for daily hassles that may disturb a person over acute scale. This part in later stages was excluded from the final questionnaire since quantification done by the two scales was not very accurate and there were certain flaws in the scoring and basic design.

Part C included questions based on which people can be grouped in the categories of stressors namely social stressor which included disturbance in social relations at home, working place or with peer, personal stressor which included personal dissatisfaction on multitude of spheres including present job, social status, financial status, achievements and personality and finally others stressors. This part was also excluded from the final questionnaire since qualitatively identifying the stressor was very difficult as the stress faced by each subject was a combination of different stressors. Moreover, our study was aimed at finding general stress bio-markers, so, we did not require a priori grouping of subjects based on stressors at current stage.

In the final questionnaire, Part C was appended by a small set of questions which included; a) duration of physiological or psychological symptoms faced by the subject, b) information about any severe disease, subject had been afflicted with, at the time of blood collection, and c) personality traits. Thus, the final questionnaire inculcated in it; Part A and a general briefing about the subject (Table S1) to evaluate the theoretical stress status of individuals.

### Scoring Method and Analysis

**Figure 1:**
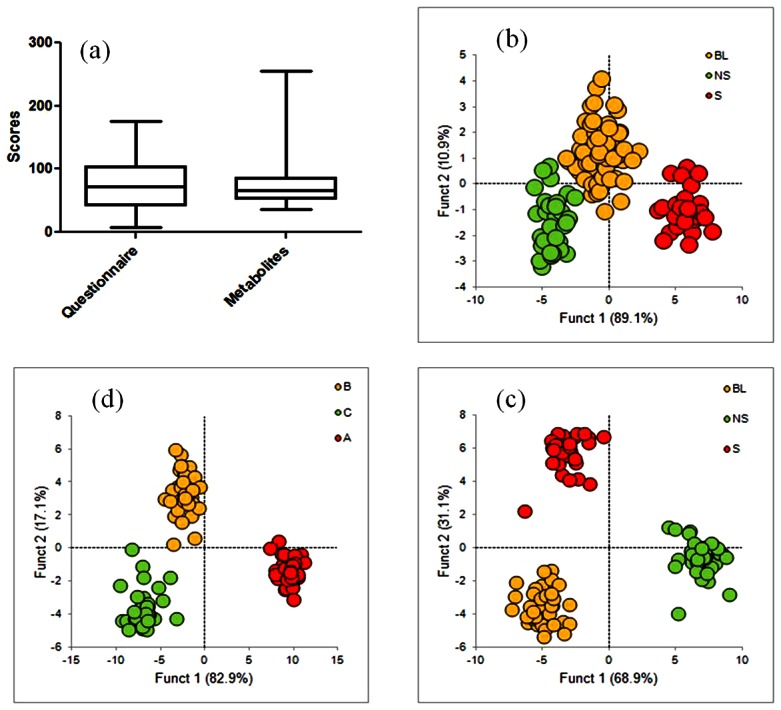
Questionnaire and metabolite scores and profiles. a) Box-whisker plots of questionnaire and metabolite scores of individuals, discriminant analysis of b) questionnaire and c) metabolites based profiles where individuals were assigned to S, NS, BL groups on the basis of questionnaire scores, d) Discriminant analysis of metabolite based profiles based on metabolite scores as derived from distribution plots where distributions were termed as A, B and C.

For questionnaire analysis scoring (Q-score) was done on a scale of 0 to 4. For metabolite analysis scoring (M-score) was determined by taking sum of the areas under the normalized peaks. Relative distribution of the scores for individuals from both analyses was plotted either as scatter or Box-Whisker plot. Cumulative score above or below 1-SD was apriori grouped as stressed (S) or Non-stressed (NS), respectively. Values within 1-SD are termed as borderline (BL). The ranges of scores for respective groups are mentioned in Table S2 with respective plots shown in figure 1a.

### 2-Dimensional Gel Electrophoresis

All materials, unless mentioned otherwise, were obtained GE Health Care, USA. IsoElectric Focusing (IEF) was performed using an IPGPhor electrophoresis unit (GE Health Care, USA) following the methods described in the instruction manual. Immobiline Dry Strips (pH 3–10, IPG strip 24 cm, 13 cm from GE Health Care) were used for separation in the first dimension. Serum samples were chosen randomly for initial standardization. A total of 500 µg of total protein was analyzed for each sample to achieve adequate resolution to identify protein spots on the gels for comparison. For IEF, aliquots of serum samples containing 500 µg and 400 µg of total protein were prepared in 450 µL and 250 µl of rehydration buffer, respectively, containing 8M urea, 0.5% (w/v) CHAPS, 0.2% (w/v) DTT, 0.5% (v/v) IPG buffer, 0.002% bromophenol blue for 24 cm and 13 cm strips, respectively and were incubated with IPG strips for 12 h. IEF was conducted at 20°C using the following voltage gradient: For 24 cm strip: Step 150 V, 1 hr; Gradient 500 V, 3 hr; Gradient 2000 V, 4 hr; Gradient 5000 V, 3 hr; Gradient 8000 V, 1 hr; Step 8000 V, 1 hr; Step 10000 V, 1 hr; Step 500 V, 3 hr. For 13 cm strip: Step 150 V, 1 hr; Gradient 500 V, 3 hr; Gradient 2000 V, 4 hr; Gradient 5000 V, 3 hr; Gradient 8000 V, 1 hr; Step 8000 V, 1 hr; Step 500 V, 3 hr. The last step was optional in both the cases. After focussing, the strips were incubated in equilibration buffer [10 mL consisting of 50 mM Tris-HCl, pH 8.8, 6M urea, 30% (v/v) glycerol, 2% SDS, bromophenol blue and 0.065 M DTT] for 15 min on a rocking platform. The strips were subsequently incubated with the same equilibration buffer substituting 10 mM iodoacetamide for DTT to alkylate cysteine sulfhydryls and were then placed on top of a 12% SDS-PAGE gel (26×20 cm) and (14×15 cm) for 24 cm and 13 cm strips, respectively.

Second dimension separation was performed in Ettan Dalt 2D-system and Ruby (GE Healthcare, USA) at 25°C for 18 h at 5 mA/gel area. A buffer consisting of 25 mM Tris-HCl, 192 mM glycine and 0.1% SDS was used. Gels were stained using silver staining method compatible with MALDI-TOF as mentioned elsewhere [22].

### Protein Gel Imaging and Analysis

After destaining, gels were scanned with a white light scanner and images were saved for further analysis. Analysis of the variation of protein expression in different samples was done using the 2D Platinum gel image master 7.0 (GE Healthcare, USA) after scanning the gels. The relative volume (referred to as intensity in the text) of each spot relative to the total volume of all spots scanned for each gel was generated by the software to correct for differences in gel staining [23]. Since, the total protein loaded was the same for all samples, no further normalization of absolute intensities of the protein spots were necessary. The significance of spot intensities were calculated using 2-way analysis of variance (ANOVA) and significant protein spots were excised and characterized by mass spectrometry as described below.

### ^1^H NMR

1D- ^1^H NMR spectra of the serum samples were recorded at a resonance frequency of 400 MHz on a Bruker Avance-400 spectrometer (Rheinstetten, Germany). Prior to NMR analysis, serum samples (540 µl) were diluted (10% ^2^H_2_0 v/v) and placed in AMEX Round Bottom MINIPUL NMR Sample Tubes (Norell Inc., USA). Water suppression using excitation sculpting with gradients was used to accomplish efficient suppression of water resonance in the spectral data [24]. For each sample, 64 free induction decays were accumulated over a spectral width of 7183.908 Hz and at a temperature of 289.4 K and data were collected and processed for further analyses as described before [25], [26]. The acquired 1D-NMR spectra were analyzed using Mestrenova. All the peaks in spectra were integrated with reference to constant peak at around 5.32 ppm which was set to zero integral. The peaks' integrated area or the intensity were then used for multivariate discriminant analysis. The spectral profile was similar to that of published ^1^H-NMR profile obtained from human serum samples [27] and the peaks were assigned to specific metabolites by matching the peak list with online Human Metabolome database (HMDB), http://www.hmdb.ca/ and annotating them accordingly.

### Statistics

Student's t test was used for single comparisons. Two-way ANOVA was used for multiple comparisons. Statistical significance was assumed for p<0.05.

### Power Calculation

Power calculation for sample size determination to yield statistically significant results was performed using G*power version 3.1.3 [28]. To attain a power of 0.95 with effect size 0.7 for α = 0.05 sample size for total population was required 106 ( = N). In the current study we analyzed data from 124 individuals.

### Discriminant Analysis

Discriminant analysis was performed on normalized values of (a) protein spot intensities (% vol) from 2DE gels of various identified proteins, (b) peak intensities of various identified metabolites as described in a previous section and (c) scores of cumulative questionnaire markers. These normalized values for the variables are termed as abundance or normalized abundance for future reference in rest of the manuscript. Normalization of data for each methodology was done separately in Genespring software (Agilent Inc., USA) with respect to the median value of each variable from control treatment. Grouping of data, with the normalized values to identify the distribution pattern for select conditions, was done by discriminant analysis to calculate the linear combinations of the variables such that one discrminant function (Fn) is orthogonal to the other. Two-dimensional orthogonal Eigen value-scores for all variables for each individual in a group were calculated using StatistiXL package (add in to Microsoft Excel) in order to identify the pattern or cluster in the data as a result of the stress condition using serum samples collected from individuals consented for this study.

### Pathway-Metabolite Network

We generated the pathway-metabolite network in cytoscape version 2.8.3 using the following strategy. Using the pathway and metabolite names as node attributes a ‘sif’ file was generated, , where each metabolite that populated a pathway was connected to the pathway through an edge. The pathway nodes were given a score based on their population by the metabolites, while the metabolite nodes were given a score based on their mean experimental abundance in the group they represent. This score was loaded into the sif network as ‘pval’ file and was used for generating node sizes proportional to respective scores or values. Finally, using Vizmapper, discrete mapping property nodes were assigned with specific shapes and colors associated based on the values.

## Results

Figure 1a shows the box-whisker plot of Q- and M-scores calculated from questionnaire and NMR data. Score distribution for both methodologies was grouped into three as Stressed (S), Non-Stressed (NS) and Borderline (BL) following the procedure described in materials and methods. Discriminant analysis was done on these grouping to confirm the classification. Figures 1b and 1c showed discriminant analysis of classification based on Q-scores for both questionnaire and metabolite data. We, however, wanted to check if grouping based on Q-scores that was used to classify metabolite profile too was correct or not. We did a discriminant analysis of the data by grouping metabolite data independently. Figure 1d shows discriminant plot for metabolite scores grouped as A, B and C. Comparison of figure 1c and figure 1d showed similar profile and trend, irrespective of grouping scores either by Q- or M-score profile.

**Figure 2:**
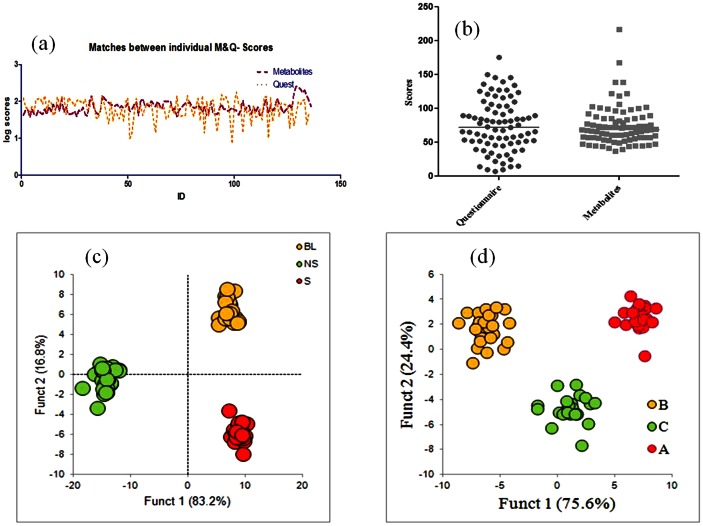
Comparison of questionnaire and metabolite data. a) Variation in normalized scores between questionnaire and metabolite scores for each individual (n = 124), b) Scatter plots of questionnaire and metabolite scores of select individuals (n = 82). Selected individuals include the subjects which had similar variations in their metabolite and questionnaire score patterns, discriminant analysis of c) questionnaire and d) metabolite based profiles where individuals were assigned to S, NS, BL groups and A, B and C groups on the basis of questionnaire score and metabolite scores respectively.

The next step was to check the pattern followed by Q-scores and M-scores for an individual with respect to rest of the population. This study was done with two purposes; 1) check the association of M-scores and Q-scores for an individual, and 2) remove outliers from the group with no correspondence between the two scores. Figure 2a and 2b show the correlation and matches in the patterns of scores for 124 individuals, respectively. It is clear that there is a positive association between Q-scores and M-scores for major population (82 individuals out of 124). Positive association means that a high Q-score corresponds to high M-score for an individual. Given the matches amongst individuals, 82 subjects were chosen for further analysis. Again a double blind study was performed for questionnaire (figure 2c) and metabolite (figure 2d) profiles with grouping based on distribution plots for the selected group of individuals. It is clear that after removing outliers from the group, the grouping becomes tighter with increased contribution of each function to the distinctiveness of the groups. It is important to note that even after removing individuals from the initial group (124 subjects), the scoring limits for both Q and M-profiles remain similar (Table S3). This observation implies robustness of scoring method as well as the consistency in data after removing outliers.

**Figure 3:**
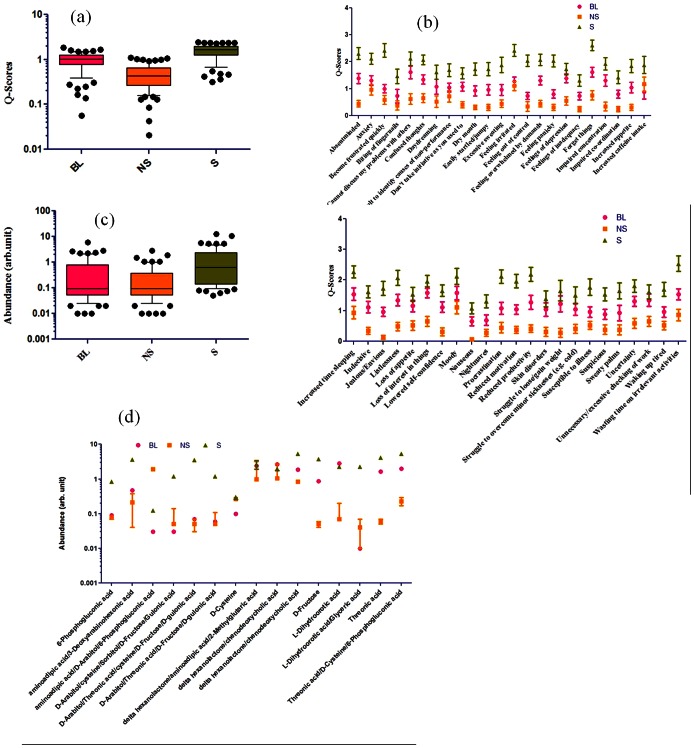
Q- and M-markers. a) Box-Whisker plot to show relative differences in the scores of questions in the questionnaire to which three groups responded to. This analysis was used for finding out significant Q-markers. b) Q-score distribution among three groups BL, NS and S for significant (p<0.05) Q-markers are shown in two panels (top and bottom), c) Box-Whisker plots to show relative differences in metabolite scores among groups representing BL, NS, and S, d) Relative differences in abundance of significant metabolites among S, NS, and BL groups.

Thus, with the background that stressed individuals show a distinct molecular and questionnaire profile pattern, we wanted to find out the contributing factors for the uniqueness of respective groups. All the statistical tests were performed while assuming a Gaussian distribution for the data which was validated by Kolmogrov Smirnov test [29]. To find out significant metabolite and questionnaire markers, 2-way ANOVA was done. Also independent group based profiles were compared using column statistics to see overall changes in the respective profiles within a group. Figure 3a shows relatively high Q-scores for stress (S) group unlike NS and BL groups, with NS individuals scoring minimum. Figure 3b (in two panels) shows the set of significant Q-markers for three groups. For NS and BL groups, there were only two questions that gave a p value less than 0.05; for BL and S groups, 6 questions (Q-markers) shows a statistical significance of p<0.05; but unlike the two comparisons, between NS and S groups; 45 questions out of 75 came up as significant Q-markers. The list of Q-markers and their scores for respective groups is given in table S3A and annotations of Q-markers are shown in table S3B. Similarly, significant metabolites were screened for metabolite profiles. Figure 3c reveals that the stressed (S) group is significantly different in terms of elevated expression of metabolites compared to other two groups (NS and BL). Figure 3d shows that the 30 significant metabolite peaks, out of a total of 187 peaks from NMR data, contributed to the differences among three groups (S, NS and BL). When the metabolites corresponding to these peaks were checked for any association with stress, it was found that out of 41 metabolite peaks, 18 metabolites were reported in literature to be related with PS or PS associated diseases. Table S4A gives a detail of these metabolites. Since, the up-regulation of these metabolites in most of the cases is associated with PS, the group with higher expression of these metabolites was assigned as stressed group. Thus, A group was assigned to be stressed, B to be borderline and C to be Non-stressed. All metabolites, mentioned in Table S4B, have been associated with PS or PS associated diseases which include liver dysfunction, IFD, metabolic syndrome (Table S5). Thus, changes in metabolite profiles point to systemic variation incurred by PS in the body.

**Figure 4:**
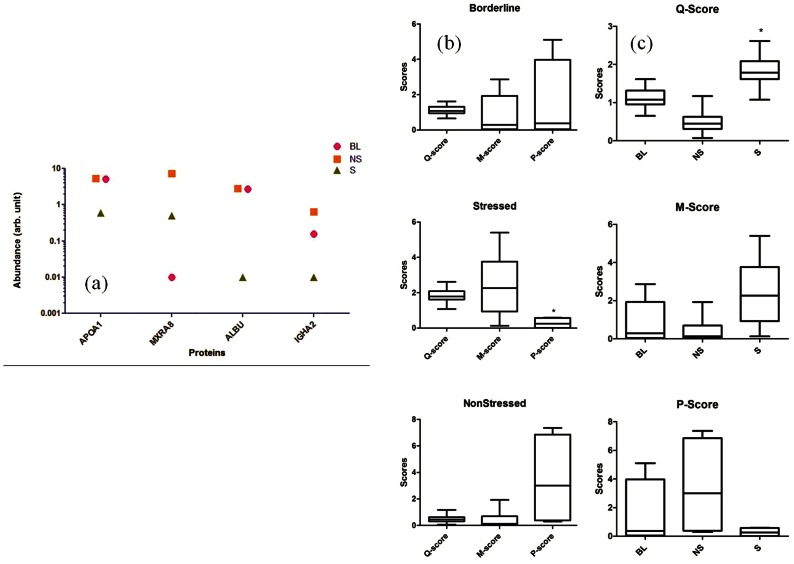
Protein Markers and comparative analyis. a) Significant (p<0.05) protein markers that show consistent differences amongst the individuals of S, NS and BL groups, b) Box-Whisker plots for different (Q-, M- and P-) scores among three groups and c) relative spread of three different groups in terms of Q-, M- and P-scores.

Finally, to add to the robustness and validity of group specific changes based on stressed profiles of individuals, proteomic analysis was done. In proteomic analysis, out of a total of 30 spots identified as different between any two groups, 8 were found significantly different across three groups. Figure 4a shows 4 proteins, out of 8 significant proteins, that have been reported to be associated with psychological stress. For example, HPT and ALBU have been reported to be down-regulated in case of PS. Although, reports related to up- or down-regulation of HPT are contradictory, ALBU has been reported to be down-regulated in humans when experience chronic stress as is also observed in the current study. Table S5 lists all significant metabolites and proteins that are associated with PS and PS dependent diseases.

Overall analysis of three scores, obtained from questionnaire, metabolite and protein profiles for stressed, non-stressed and borderline conditions, revealed that Q- and M-scores for various conditions are indistinguishable while protein data significantly stands out (figure 4b). Figure 4c further validates it by establishing the fact that Stress group significantly stands out as distinct from other two groups.

## Discussion

Current study has evaluated the effectiveness of a combination of questionnaire and molecular profiling based assessment of PS in a random group of individuals.

The use of heterogeneous group presents its limitations as well as advantages in context of our study. Unlike the current report, most of the other studies on psychological stress are done with population which is grouped with similarity in age, gender, ethnicity, and economic stature [30], [31], [32]. While apparently these studies seem alright but one major disadvantage is that the variation among individuals in a group may not be picked out with confidence. On the contrary, a heterogeneous (where dynamic range in terms of age, gender etc. is varied) distribution of individuals grouped based on stress status has the advantage of selecting a stress associated marker with statistical significance irrespective of other parameters. Such markers will be more robust and stable. In the current report, we aimed at estimating stress in a population which is not grouped on an adhoc basis of any parameter but stress. Finally, what we found that such grouping based on stress status evaluation by questionnaire matched with different experimental observations. In fact, our grouping is so stable that the serum cortisol values further supported it (Figure S1).

We have compared grouping based on Q- and M-scores independently, and it was observed that both methodologies grouped individuals similarly. The questionnaire based distribution was in fact shown to be quite effective in terms of clustering patterns observed in Q-score based metabolite grouping. This was further validated by cohort study in which 124 subjects were analyzed based on their questionnaire profiles and grouping was maintained across metabolite profiles of subjects classified based on Q-scores. Thus, our single blind study gave primary hints of the positive correlation of Q-scores with stress levels which in turn were associated with group specific metabolite profiles.

Double blind study on the other hand investigated the independent correlation of metabolite scores with stress levels. This was essential in two ways; 1) a correlation between M-scores and stressed states will allow a quick evaluation, thus easing down the analysis for regular application, 2) will give an idea of total metabolite changes with respect to PS. The distribution of subjects into three groups, viz. A, B, and C based on independent M-scores scoring method again resulted in tight grouping patterns giving a primary hint towards association of scorings with group specific metabolite patterns..

**Figure 5:**
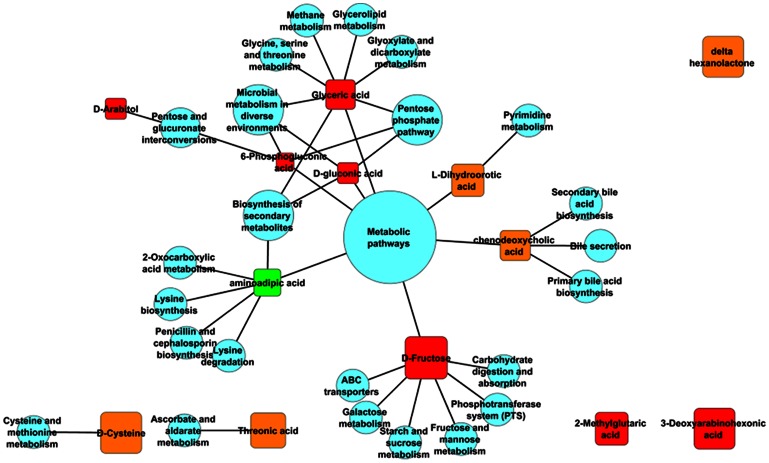
Pathway-metabolite networks. Significant metabolite biomarkers are shown as associated with directly connected pathways based on other reports. Biomarker nodes are colored differently based on experimentally determined abundance as shown in figure 3d. Size of pathway nodes are directly proportional to the number of genes populating the pathways.

Furthermore, by analyzing the significant metabolite peaks based on NMR data, 41 potential metabolites were found that could contribute to the differences between these groups. 18 out of a total of 41 potential metabolites were found to be associated either directly with PS or with PS associated disease. Interestingly, 16 of these 18 metabolites were reported to be up-regulated in diseased states [33], [34], [35], [36], [37], [38], [39], [40], [41], [42], [43], [44], [45], [46], [47], [48], [49], [50], [51], [52], [53]. This was indicative of an increase in overall abundance of metabolites associated with stress as revealed in figure 3c. Ten out of 13 metabolites (figure 3c), which were upregulated in stress (S) condition compared to BL and NS, were already reported in the literature in association to stress [54], [55], [56], [57], [58], [59], [60], [61], [62], [63], [64]. These 10 metabolites are, 6-Phosphogluconic acid, Aminoadipic acid, D-Arabitol, Cysteine, Sorbitol, D-Fructose, Threonic acid, 2-Methylglutaric acid, Chenodeoxycholic acid, L-dihydroorotic acid and these metabolites are shown to be enhanced in abundance following stress. None of these metabolites have so far been reported as a biomarker for psychological stress. However, Chenodeoxycholic acid, among the 10 metabolites found to be associated to PS in the current report, has been reported to have effects on Glucocorticoid metabolism by inhibiting the enzyme 11,beta-hydroxysteroid dehydrogenase (11β-HSD). 11β-HSD has two isoforms HSD11B1 and HSD11B2. In liver, adipose tissue, and the central nervous system HSD11B1 is highly expressed and there it reduces cortisone to the active hormone cortisol that activates glucocorticoid receptors. In kidneys, colon, salivary glands, and placenta HSD11B2 oxidizes cortisol to cortisone and prevents over-activation of the mineralocorticoid receptors We also found that, there was an overall increase in metabolite abundance for stressed group. Thus, group A was designated to be stressed group since its profiles were very similar to the ones reported in literature. This analysis gave two important insights in relation to metabolite profiles; 1) M-scores are positively correlated with stressed state, a trend similar to questionnaire profiles and therefore can be used for group assignment, 2) there is a total upregulation of metabolites associated with stressed state. All 34 metabolites when compared across the groups showed an elevated level in stressed subset (middle panel in figure 4c). Out of 34 metabolites, 6 metabolites were found to be significantly different among three groups. These included; L-alpha aminobutyric acid, Hydroxyisocaproic acid, Threonic acid, Aminoadipic acid, D-Fructose and 3-deoxyarabinohexonic acid. Out of six metabolites, elevated levels of 3-hydroxyisocaproic acid have been associated with PS mediated inflammatory bowel syndrome. The rest of five metabolites have been associated with various metabolic and hepatic disorders as well as ageing. Although there are no direct reports of their association with PS, there are reports which state that PS worsens the progression of these diseases characterized by the above stated metabolite markers. A gene network is shown (figure 5) of most populated pathways based on relative abundance of significantly different metabolites colored differently for various conditions. Size of related pathways is directly proportional with the number of genes populating the pathways. The network establishes association of experimentally determined significant metabolite biomarkers with various stress conditions.

With the preliminary idea of metabolite biomarkers, protein profiles were compared among S, NS and BL groups to check if any consistent pattern emerges and whether it can be related to metabolite results. In fact, it was found that stressed profiles again stand out in terms of total protein abundance when compared with NS and BL groups. But unlike metabolites, protein abundance is down-regulated in stressed group. Out of a total of 30 protein spots identified as potential protein bio-markers, 8 were found to be significantly different across the three groups. These included; APOA1, HPT, TTHY, MXRA8, TALDO, TRFE, ALBU, and IGHA2. Out of these 8 proteins, IGHA2, HPT, ALBU and TTHY have been associated directly with PS. Rest of 4 proteins though not directly related to PS, but have been reported to be involved in hepatic dysfunctions and atherosclerosis. Out of 4proteins associated with PS, only APOA1 has already been reported to be associated with psychological stress and it was reported that serum concentrations of APOA1 (acute phase marker) was low in severely affected patients of Systemic inflammatory response syndrome (SIRS), which is seen as an effect of chronic stress response [65]. It is clear from the current report that metabolite and protein profiles cross validate each other in terms of changes in protein expressions leading to the appearance of markers similar to these changes. For example, hepatic and metabolic disorders related protein and metabolite expressions are consistent across two profiles. This further validates the data in terms of consistency across all the profiles.

Besides metabolite and protein markers, there were some significant Q-markers associated with stressed group. The statistical analysis revealed similar difference patterns among S, NS and BL groups. The maximum question markers with statistical significance were found to be in case of S, NS population followed by S, BL (only 6 markers) and least in NS, BL (only 2 markers). This clearly goes well with the trend observed in metabolite and protein profiles with greatest difference between S, and NS groups. It is, therefore, very interesting to note that differences between S and BL as well as NS and S group remain significantly different in questionnaire, metabolite and protein profiles but NS and BL profiles do not vary much from each other. In questionnaire profiles, the question markers, which were key to the differences between groups correspond to both physiological and psychological aspects, include anxiety, feeling of depression, disinterested or disinclined to do things, moody, jealous based on psychological perspective. From physiological perspective examples include excessive sweating of palms and hands, increased or decreased appetite, struggle to overcome minor sickness, dry mouth. All the factors have been correlated with PS and hence justify the assessment by the questionnaire. Furthermore, the analysis of contribution of each profile to the PS assessment hints towards the applicability of questionnaire as a quick and easy method of routine evaluation.

It is perhaps clear from the above discussion that in stressed subjects, certain changes are initiated at the molecular level as well as neurological level in terms of perception of a situation as evident from metabolite- protein and questionnaire profiles respectively. These markers give us an indication of basic changes incurred in the functioning of body by PS. A consistency in the patterns of up-regulation or down-regulation of molecular profiles makes stressed group distinctive of NS and BL groups. Moreover, following similar trends in physiological regulation, two profiles corroborate each other in terms of the biology of stress manifestation, which further add up to the reliability of current results and methodologies. Questionnaire profiles also match with the trend observed in molecular profiles as well as highlighting changes perceived by stressed individuals at both psychological and physiological level. Thus, all the profiles imply a change at systemic levels of a stressed individual which might result in its predisposition to diseases.

## Supporting Information

Questionnaire S1 **Questionnaire document for evaluation of stress status of individuals.**
(DOCX)

Table S1 **Alternate Part C of the Questionnaire.**
(DOC)

Table S2 **Scoring method and values.**
(DOC)

Table S3 **Questionnaire IDs and references.**
(DOC)

Table S4 **Metabolite IDs and references.**
(DOC)

Table S5 **Significant metabolites and proteins.**
(DOC)

Figure S1 **Absolute cortisol levels for three groups (BL, NS and S) are shown.** Error bars shown are ±1SD of the data. Statistical significance between groups is also shown.
(TIF)
