# Supplementary material for: Estimation of Psychological Stress in Humans: A Combination of Theory and Practice
Source: PLoS One. 2013 May 15;8(5):e63044. doi: 10.1371/journal.pone.0063044 (PMC3654918; doi:10.1371/journal.pone.0063044)
Supplement: Questionnaire S1 — Questionnaire document for evaluation of stress status of individuals. (DOCX) [file pone.0063044.s001.docx]

Please note that the following questionnaire is part of our ongoing research project on stress and Metabolic Syndrome (SMS) in School of Biological Sciences, NISER under the supervision of Dr. Palok Aich ([palok.aich@niser.ac.in](mailto:palok.aich@niser.ac.in)). We would like you to answer the following questions as best as you could so that we could use it as a basis of correlating stress with various associated diseases at the molecular, cellular and genetic level.

Estimated time to complete this questionnaire is 20 minutes. We sincerely thank you for your assistance and co-operation. Your information will remain anonymous, however you will be given a computer generated ID, please bring it when you will be called on for donating blood for further research on stress by our group.

Please check if you give consent to donate your blood for research.

I would like to volunteer to donate blood for this research Yes No

# Objectives

1. Stress estimation
2. Factors involved in stressed state (for clustering of people based on common factors)

# Idea

A set of standard questionnaire to measure stress followed by questions on the basis of which the people can be grouped

How would I like to group people?

On the basis of:

1. Stressed state: intensity of stress, based on stress questionnaire
2. Chronic and acute stress: directly ask in one of the questions or through questionnaire
3. Stressors:
   - 1. *Social causes:*
        1. Relations at home and family
        2. Relations at working place
        3. Relations with peer: may involve people from different stages of life, may or may not be far.
        4. Relations with socializing group: present group of people with which one interacts.
     2. *Personal causes:*
        1. Not satisfied with the present job/education
        2. Not satisfied with the social status
        3. Not satisfied with the financial status
        4. Not satisfied with the achievements
        5. Not happy with your personality , feeling of unhealthiness, not capable of carrying oneself, lack of self confidence
        6. Lack certain qualities like time management, self-organization, self-discipline
        7. Marital status
        8. Has suffered a big trauma or a set back
        9. Worried about future
     3. *Other causes:*
        1. Pressure from family on certain things like marriage, job, income
        2. Staying away from home for quite long
        3. Recently left home for the first time

**PART A**

The underneath given questionnaire will measure the stress in the patients by taking into consideration both psychological and physiological changes that have taken place during stressed state.

Rate the parameters mentioned below according to given scores:

NEVER=0, SELDOM=1, SOMETIME=2, OFTEN=3, REGULAR=4

| Heart pounding or racing | 0 | 1 | 2 | 3 | 4 |
| --- | --- | --- | --- | --- | --- |
| Trembling/shaking | 0 | 1 | 2 | 3 | 4 |
| Grinding of teeth (even in your sleep) | 0 | 1 | 2 | 3 | 4 |
| Do not sleep well | 0 | 1 | 2 | 3 | 4 |
| Susceptible to illness | 0 | 1 | 2 | 3 | 4 |
| Stomach pains | 0 | 1 | 2 | 3 | 4 |
| Headaches | 0 | 1 | 2 | 3 | 4 |
| Migraine headaches | 0 | 1 | 2 | 3 | 4 |
| Feeling tired constantly | 0 | 1 | 2 | 3 | 4 |
| Constipation | 0 | 1 | 2 | 3 | 4 |
| Hollow stomach | 0 | 1 | 2 | 3 | 4 |
| Lowered self-confidence | 0 | 1 | 2 | 3 | 4 |
| Loss of appetite | 0 | 1 | 2 | 3 | 4 |
| Excessive sweating (e.g. hands, face, arm | 0 | 1 | 2 | 3 | 4 |
| pits, etc.) | 0 | 1 | 2 | 3 | 4 |
| Sweaty palms | 0 | 1 | 2 | 3 | 4 |
| Listlessness– don’t feel like doing stuff | 0 | 1 | 2 | 3 | 4 |
| Forget things | 0 | 1 | 2 | 3 | 4 |
| Sweaty palms | 0 | 1 | 2 | 3 | 4 |
| Listlessness– don’t feel like doing stuff | 0 | 1 | 2 | 3 | 4 |
| Forget things | 0 | 1 | 2 | 3 | 4 |
| Absentminded | 0 | 1 | 2 | 3 | 4 |
| Feeling irritated | 0 | 1 | 2 | 3 | 4 |
| Nauseous | 0 | 1 | 2 | 3 | 4 |
| Considered suicide | 0 | 1 | 2 | 3 | 4 |
| Pessimistic | 0 | 1 | 2 | 3 | 4 |
| Jealous/Envious | 0 | 1 | 2 | 3 | 4 |
| Moody | 0 | 1 | 2 | 3 | 4 |
| Pain in the lower back | 0 | 1 | 2 | 3 | 4 |
| Feelings of depression | 0 | 1 | 2 | 3 | 4 |
| Anxiety | 0 | 1 | 2 | 3 | 4 |
| Loss of interest in things | 0 | 1 | 2 | 3 | 4 |
| Sensitive and/or Touchy | 0 | 1 | 2 | 3 | 4 |
| Muscle pain | 0 | 1 | 2 | 3 | 4 |
| Indecisive | 0 | 1 | 2 | 3 | 4 |
| Unnecessary/excessive checking of work | 0 | 1 | 2 | 3 | 4 |
| Difficulty with breathing | 0 | 1 | 2 | 3 | 4 |
| Struggle to overcome minor sicknesses | 0 | 1 | 2 | 3 | 4 |
| Suspicious | 0 | 1 | 2 | 3 | 4 |
| Hair loss | 0 | 1 | 2 | 3 | 4 |
| Throat irritations | 0 | 1 | 2 | 3 | 4 |
| Lost sense of humor | 0 | 1 | 2 | 3 | 4 |
| Impaired concentration | 0 | 1 | 2 | 3 | 4 |
| Struggle to loose/gain weight even when following a diet | 0 | 1 | 2 | 3 | 4 |
| Heartburn | 0 | 1 | 2 | 3 | 4 |
| Skin disorders | 0 | 1 | 2 | 3 | 4 |
| Don’t take initiative as you used to | 0 | 1 | 2 | 3 | 4 |
| Nightmares | 0 | 1 | 2 | 3 | 4 |
| Dry mouth | 0 | 1 | 2 | 3 | 4 |
| Consume tonics | 0 | 1 | 2 | 3 | 4 |
| Diarrhea | 0 | 1 | 2 | 3 | 4 |
| Nervous twitches in face or scalp | 0 | 1 | 2 | 3 | 4 |
| Feelings of inadequacy | 0 | 1 | 2 | 3 | 4 |
| Easily startled/jumpy | 0 | 1 | 2 | 3 | 4 |
| Increased appetite | 0 | 1 | 2 | 3 | 4 |
| Impaired co-ordination | 0 | 1 | 2 | 3 | 4 |
| Uncertainty | 0 | 1 | 2 | 3 | 4 |
| Become frustrated quickly | 0 | 1 | 2 | 3 | 4 |
| Less involvement with others | 0 | 1 | 2 | 3 | 4 |
| Biting of fingernails | 0 | 1 | 2 | 3 | 4 |
| Reduced motivation | 0 | 1 | 2 | 3 | 4 |
| Increased caffeine intake (coffee, tea, coke, etc.) | 0 | 1 | 2 | 3 | 4 |
| Restlessness | 0 | 1 | 2 | 3 | 4 |
| Poor judgment | 0 | 1 | 2 | 3 | 4 |
| Increased smoking | 0 | 1 | 2 | 3 | 4 |
| Feeling out of control | 0 | 1 | 2 | 3 | 4 |
| Confused thoughts | 0 | 1 | 2 | 3 | 4 |
| Increased time sleeping | 0 | 1 | 2 | 3 | 4 |
| Use tranquilizers, sleeping pills | 0 | 1 | 2 | 3 | 4 |
| Waking up tired | 0 | 1 | 2 | 3 | 4 |
| Feeling overwhelmed by demands | 0 | 1 | 2 | 3 | 4 |
| Excessive blinking | 0 | 1 | 2 | 3 | 4 |
| Daydreaming | 0 | 1 | 2 | 3 | 4 |
| Procrastination | 0 | 1 | 2 | 3 | 4 |
| Feeling panicky | 0 | 1 | 2 | 3 | 4 |
| Reduced productivity | 0 | 1 | 2 | 3 | 4 |
| Wasting time on irrelevant activities | 0 | 1 | 2 | 3 | 4 |
| Cannot discuss my problems with others | 0 | 1 | 2 | 3 | 4 |
| Difficult to identify causes of non-performance | 0 | 1 | 2 | 3 | 4 |

**PART B**

Holmes and Rahe scale for stress induced by life changing events. It measures the stress over a period of two years (chronic stress). The conditions mentioned should be considered for stress before and after their occurrence.

**Holmes and Rahe Stress Questionnaire**

# For people of age group above 26 years

| **Life event** | **Life change units** |
| --- | --- |
| Death of a spouse | 100 |
| Divorce | 73 |
| Marital separation | 65 |
| Death of a close family member | 63 |
| Personal injury or illness | 53 |
| Marriage | 50 |
| Dismissal from work | 47 |
| Marital reconciliation | 45 |
| Retirement | 45 |
| Change in health of family member | 44 |
| Pregnancy | 40 |
| Sexual difficulties | 39 |
| Gain a new family member | 39 |
| Business readjustment | 39 |
| Change in financial state | 38 |
| Death of a close friend | 37 |
| Change to different line of work | 36 |
| Change in frequency of arguments | 35 |
| Major mortgage | 32 |
| Foreclosure of mortgage or loan | 30 |
| Change in responsibilities at work | 29 |
| Child leaving home | 29 |
| Trouble with in-laws | 29 |
| Outstanding personal achievement | 28 |
| Spouse starts or stops work | 26 |
| Change in living conditions | 25 |
| Revision of personal habits | 24 |
| Trouble with boss | 23 |
| Change in working hours or conditions | 20 |
| Change in residence | 20 |
| Change in recreation | 19 |
| Change in social activities | 18 |
| Minor mortgage or loan | 17 |
| Change in sleeping habits | 16 |
| Change in number of family reunions | 15 |
| Change in eating habits | 15 |
| Vacation | 13 |
| Festival | 12 |
| Minor violation of law | 11 |

# For people of Age group 18-26 years

| **Life Event** | **Life Change Units** |
| --- | --- |
| Getting married | 95 |
| Death of parent | 100 |
| Acquiring a visible deformity | 80 |
| Divorce of parents | 90 |
| Becoming involved with drugs or alcohol | 50 |
| Marital separation of parents | 69 |
| Death of a brother or sister | 68 |
| Change in acceptance by peers | 67 |
| Death of a close friend | 63 |
| Having a visible congenital deformity | 62 |
| Serious illness requiring hospitalization | 58 |
| Failure of a grade in school | 56 |
| Not making an extracurricular activity | 55 |
| Hospitalization of a parent | 55 |
| Breaking up with boyfriend or girlfriend | 53 |
| Beginning to date | 51 |
| Suspension from school | 50 |
| Birth of a brother or sister | 50 |
| Increase in arguments between parents | 47 |
| Loss of job by parent | 46 |
| Outstanding personal achievement | 46 |
| Change in parent's financial status | 45 |
| Accepted at college of choice | 43 |
| Being a senior in high school | 42 |
| Hospitalization of a sibling | 41 |
| Increased absence of parent from home | 38 |
| Brother or sister leaving home | 37 |
| Begin or end school | 26 |
| Addition of third adult to family | 34 |
| Decrease in arguments between parents | 27 |
| Decrease in arguments with parents | 26 |
| Mother or father beginning work | 26 |

# For measuring acute stress

This is a tool to measure the amount of stress you experience in your daily life. 66 statements are included. Read slowly and carefully, state how far it is true in your case. You may please choose any one of the following five options to each item.

A. Very true, I agree fully
B. True, I agree
C. I cannot say
D. Not true, I disagree
E. Not true, I totally disagree

1. I do things in a hurry.
2. I like to travel slowly.
3. I eat food faster.
4. I never interrupt when others talk.
5. I want to finish works with neatness and perfection.
6. I speak slowly.
7. Seeing lazy people I get angry.
8. I never bet with others.
9. I feel tensed on thinking about my responsibilities.
10. I am not happy to wait in a queue.
11. I always consider the feelings of others while talking.
12. I take intoxicants.
13. I pray regularly.
14. I am interested in religious books.
15. My sexual life is not satisfactory.
16. I watch movies and plays.
17. I practice meditation.
18. I don’t reveal secrets to others.
19. I can’t stay away from home.
20. I fell tensed on unexpected arrival of a guest.
21. I feel disturbed on an unexpected expenditure.
22. I have debts.
23. I quarrel frequently with spouse.
24. I feel that some of my family members are against me.
25. I am not properly understood.
26. I feel devaluated in society.
27. I can’t plan my financial budget properly.
28. I am not being loved.
29. I have a lot of family problems.
30. I reach home late.

31. I discuss my problems with family members.
32. I have experiences of loosing job unexpectedly.
33. I am often scolded by superiors for coming late.
34. I fear my work place.
35. I quarrel with colleagues.
36. I had been a scapegoat in fight between superiors.
37. I don’t like in engaging in love affairs.
38. I receive confusing and contradicting instructions from above.
39. My job is boring one.
40. I am not paid adequately.
41. My work evokes prick of conscience.
42. I had to bear injustice silently.
43. I am satisfied at work.
44. I am well trained for my work.
45. I get angry soon.
46. I don’t loose opportunities to help others.
47. I believe and spread rumors.
48. I am a patient listener.
49. I am willing to accept my faults.
50. At times I feel like destroying everything.
51. I suffer from headache.
52. I have frequent attacks of chest pain.
53. I have poor appetite.
54. I sweat without reason.
55. I don’t get adequate sleep.
56. I have nightmares.
57. I loose control soon.
58. I hate criticism.
59. I feel anxious.
60. I feel calm.
61. I usually regret for what has happened.
62. I fell sorry.
63. I feel frustrated.
64. I am happy.
65. I am confident.
66. I feel worthless.

**Part C**

- **ID**
- **Age**
- **Sex**
- **Personality traits**:
- Lazy or active
- Introvert or extrovert
- Reserved or gregarious
- Easy going or anxious
- Restless or patient
- Pessimistic or optimistic
- **Marital Status**
  - *If married,*
    - - How many children do you have?
      - How many members of your family stay with you presently
      - How much time do you spend with them?
- Quite a lot
- Sufficient enough
- Little
- Very little
- Not at all
  - - - Do you enjoy this time?
- Very much
- To certain extent
- Very little
- Not at all
  - - - How much time do you spend with family members other than the one in immediate relation?
- Quite a lot
- Sufficient enough
- Little
- Very little
- Not at all
  - - - How do you feel during this time?

Very good

Good

Ok

Not good

Not good at all

- - *If unmarried*
- How often do you stay at home with your family?
- Always
- Quite often
- Often
- Occasionally
- Not at all
- How do you feel about this time?
- Very good
- Good
- Ok
- Not good
- Not good at all
- **Education:**
- **Native place:**
- *If not a native*
  - - For how long you are staying away from home?
    - How strongly you feel like going back to your home town?
      - Very strongly
      - Strongly
      - Not strongly
      - Not at all
- **Occupation:**
  - - - For how long you have been working?
      - How you categorize your present work?

Easy going

Optimal work

Consistently high pressure work

Seldom high pressure work

- Are you satisfied with your present job in terms of:
  - - - - Income? Yes/No
        - Working group? Yes/No
        - Ambience? Yes/No
        - Personal time? Yes/No
        - Job status? Yes/No
- Would you like to change your job?
- Yes, in all the possibilities
- Yes, if I get a suitable one
- No, I don’t need to change urgently
- No, It’s not needed at all
- **Social relations**
  - Do you socialize with people?
    - Always
    - Quite often
    - Often
    - Occasionally
    - Not at all
- Do you contact your peers?
  - Always
  - Quite often
  - Often
  - Occasionally
  - Not at all
- **Personal**
  - Do you want to improve yourself:
    - Strongly in all aspects
    - Strongly in few aspects
    - Not so strongly
    - Not at all
- Do you seek for others help in your work?
  - Always
  - Quite often
  - Often
  - Occasionally
  - Not at all
- How do you manage yourself and your time?
  - Very well, I always finish all my work in time
  - Quite well, I often finish my work in time
  - Not very well, I lag in deadlines
  - Not at all, I never finish my work at time
- **Health**
  - Do you suffer from any kind of severe illness? Yes/ No
  - How do you feel about it?
    - I can cope well with it
    - It disturbs me a lot sometimes
    - I am consistently disturbed
    - I want to get rid in any possible way
- Have you started taking drugs and alcohol? Yes/ No
- Have you changed your dietary habits? Yes/ No
- What kind of food habits you have shifted to?
  - High fat, low nutrition
  - High fat, high nutrition
  - Low fat, low nutrition
  - Low fat, high nutrition
- Have you introduced new tasks in your routine in recent past like:
  - Morning walk/ exercise
  - Yoga/ meditation
  - Praying or attending a religious assembly
  - Working for other people in help
- Have you become susceptible to any diseases recently (less than year? Yes/ No
- **According to you, for how long, the period any kind of health related (both mental and physical) disturbance if any has been there in your life?**
