# Supplementary material for: Estimation of Psychological Stress in Humans: A Combination of Theory and Practice
Source: PLoS One. 2013 May 15;8(5):e63044. doi: 10.1371/journal.pone.0063044 (PMC3654918; doi:10.1371/journal.pone.0063044)
Supplement: Table S1 — Alternate Part C of the Questionnaire. (DOC) [file pone.0063044.s002.doc]

**Table S1.** Summary of scores (as mentioned below) associated with important questions leading to evaluate the overall stress status (termed as general briefing in the main text)^*^

NEVER=0, SELDOM=1, SOMETIME=2, OFTEN=3, REGULAR=4

| Heart pounding or racing | 0 | 1 | 2 | 3 | 4 |
| --- | --- | --- | --- | --- | --- |
| Trembling/shaking | 0 | 1 | 2 | 3 | 4 |
| Grinding of teeth (even in your sleep) | 0 | 1 | 2 | 3 | 4 |
| Do not sleep well | 0 | 1 | 2 | 3 | 4 |
| Susceptible to illness | 0 | 1 | 2 | 3 | 4 |
| Stomach pains | 0 | 1 | 2 | 3 | 4 |
| Headaches | 0 | 1 | 2 | 3 | 4 |
| Migraine headaches | 0 | 1 | 2 | 3 | 4 |
| Feeling tired constantly | 0 | 1 | 2 | 3 | 4 |
| Constipation | 0 | 1 | 2 | 3 | 4 |
| Hollow stomach | 0 | 1 | 2 | 3 | 4 |
| Lowered self-confidence | 0 | 1 | 2 | 3 | 4 |
| Loss of appetite | 0 | 1 | 2 | 3 | 4 |
| Excessive sweating (e.g. hands, face, arm pits, etc.) | 0 | 1 | 2 | 3 | 4 |
| Sweaty palms | 0 | 1 | 2 | 3 | 4 |
| Listlessness– don’t feel like doing stuff | 0 | 1 | 2 | 3 | 4 |
| Forget things | 0 | 1 | 2 | 3 | 4 |
| Absentminded | 0 | 1 | 2 | 3 | 4 |
| Feeling irritated | 0 | 1 | 2 | 3 | 4 |
| Nauseous | 0 | 1 | 2 | 3 | 4 |
| Considered suicide | 0 | 1 | 2 | 3 | 4 |
| Pessimistic | 0 | 1 | 2 | 3 | 4 |
| Jealous/Envious | 0 | 1 | 2 | 3 | 4 |
| Moody | 0 | 1 | 2 | 3 | 4 |
| Pain in the lower back | 0 | 1 | 2 | 3 | 4 |
| Feelings of depression | 0 | 1 | 2 | 3 | 4 |
| Anxiety | 0 | 1 | 2 | 3 | 4 |
| Loss of interest in things | 0 | 1 | 2 | 3 | 4 |
| Sensitive and/or Touchy | 0 | 1 | 2 | 3 | 4 |
| Muscle pain | 0 | 1 | 2 | 3 | 4 |
| Indecisive | 0 | 1 | 2 | 3 | 4 |
| Unnecessary/excessive checking of work | 0 | 1 | 2 | 3 | 4 |
| Difficulty with breathing | 0 | 1 | 2 | 3 | 4 |
| Struggle to overcome minor sicknesses (e.g. a cold) | 0 | 1 | 2 | 3 | 4 |
| Suspicious | 0 | 1 | 2 | 3 | 4 |
| Hair loss | 0 | 1 | 2 | 3 | 4 |
| Throat irritations | 0 | 1 | 2 | 3 | 4 |
| Lost sense of humor | 0 | 1 | 2 | 3 | 4 |
| Impaired concentration | 0 | 1 | 2 | 3 | 4 |
| Struggle to loose/gain weight even when following a diet | 0 | 1 | 2 | 3 | 4 |
| Heartburn | 0 | 1 | 2 | 3 | 4 |
| Skin disorders | 0 | 1 | 2 | 3 | 4 |
| Don’t take initiative as you used to | 0 | 1 | 2 | 3 | 4 |
| Nightmares | 0 | 1 | 2 | 3 | 4 |
| Dry mouth | 0 | 1 | 2 | 3 | 4 |
| Consume tonics (bioplus, liviton, lucozade, pharmaton, etc.) | 0 | 1 | 2 | 3 | 4 |
| Diarrhoea | 0 | 1 | 2 | 3 | 4 |
| Nervous twitches in face or scalp | 0 | 1 | 2 | 3 | 4 |
| Feelings of inadequacy | 0 | 1 | 2 | 3 | 4 |
| Easily startled/jumpy | 0 | 1 | 2 | 3 | 4 |
| Increased appetite | 0 | 1 | 2 | 3 | 4 |
| Impaired co-ordination | 0 | 1 | 2 | 3 | 4 |
| Uncertainty | 0 | 1 | 2 | 3 | 4 |
| Become frustrated quickly | 0 | 1 | 2 | 3 | 4 |
| Less involvement with others | 0 | 1 | 2 | 3 | 4 |
| Biting of fingernails | 0 | 1 | 2 | 3 | 4 |
| Reduced motivation | 0 | 1 | 2 | 3 | 4 |
| Increased caffeine intake (coffee, tea, coke, coke light, red bull, etc.) | 0 | 1 | 2 | 3 | 4 |
| Restlessness | 0 | 1 | 2 | 3 | 4 |
| Poor judgment | 0 | 1 | 2 | 3 | 4 |
| Increased smoking | 0 | 1 | 2 | 3 | 4 |
| Feeling out of control | 0 | 1 | 2 | 3 | 4 |
| Confused thoughts | 0 | 1 | 2 | 3 | 4 |
| Increased time sleeping | 0 | 1 | 2 | 3 | 4 |
| Use tranquilisers, sleeping pills | 0 | 1 | 2 | 3 | 4 |
| Waking up tired | 0 | 1 | 2 | 3 | 4 |
| Feeling overwhelmed by demands | 0 | 1 | 2 | 3 | 4 |
| Excessive blinking | 0 | 1 | 2 | 3 | 4 |
| Daydreaming | 0 | 1 | 2 | 3 | 4 |
| Procrastination | 0 | 1 | 2 | 3 | 4 |
| Feeling panicky | 0 | 1 | 2 | 3 | 4 |
| Reduced productivity | 0 | 1 | 2 | 3 | 4 |
| Wasting time on irrelevant activities | 0 | 1 | 2 | 3 | 4 |
| Cannot discuss my problems with others | 0 | 1 | 2 | 3 | 4 |
| Difficult to identify causes of non-performance | 0 | 1 | 2 | 3 | 4 |

**^*^Given the same set of questions, your answers to them had remained the same for:**

- Only Last 4 months
- Last 8-4 months
- Last 12-8 months

**Please check the suitable options highlighting your personality:**

- Lazy or active
- Introvert or extrovert
- Reserved or gregarious
- Easy going or anxious
- Restless or patient
- Pessimistic or optimistic

**Are you suffering from any kind of severe or frequently occurring illness since last one year?**

**Please name the disease or illness?**
