# Supplementary material for: Estimation of Psychological Stress in Humans: A Combination of Theory and Practice
Source: PLoS One. 2013 May 15;8(5):e63044. doi: 10.1371/journal.pone.0063044 (PMC3654918; doi:10.1371/journal.pone.0063044)
Supplement: Table S2 — Scoring method and values. (DOC) [file pone.0063044.s003.doc]

**Table S2:** Summary of Q- and M-scores for three studies and respective metabolite and questionnaire profiles^a^

| **Sample set, Description** | **Data** | **(Maximum Value, Minimum Value)** | **Median** | **Standard Deviation** | **First SD interval** | **Percentile (75, 25)** |
| --- | --- | --- | --- | --- | --- | --- |
| **Pilot study**  **(39)** | Questionnaire | (149, 34) | 94 | 33.46 | 29% | (121,67) |
|  | Metabolite | (299,58.07) | 125.2 | 44.86 | 18.62% | (148.48, 101.92) |
| **Cohort study (124)** | Questionnaire | (176,7) | 73.5 | 38.56 | 23% | (90,56) |
|  | Metabolite | (254.9, 36.91) | 66.83 | 38 | 17.4% | (78.48, 55.16) |
| **Screened group**  **(82)** | Questionnaire | (176,7) | 72 | 38.65 | 22.8% | (89,56) |
|  | Metabolite | (216.6, 36.91) | 68.39 | 29.35 | 16.33% | (80,57.73) |

^a^ Range here shows the interval that will be considered as BL or B group. Above the upper limit individuals were assigned as Stressed (S) group and below the lower limit individuals were assigned NonStressed (NS) group.
