# Supplementary material for: Estimation of Psychological Stress in Humans: A Combination of Theory and Practice
Source: PLoS One. 2013 May 15;8(5):e63044. doi: 10.1371/journal.pone.0063044 (PMC3654918; doi:10.1371/journal.pone.0063044)
Supplement: Table S3 — Questionnaire IDs and references. (DOC) [file pone.0063044.s004.doc]

**Table S3**

1. Question IDs and corresponding cumulative Q- and M-scores

| **ID** | **Q-Score** | **M-Score** | **ID** | **Q-Score** | **M-Score** | **ID** | **Q-Score** | **M-Score** |
| --- | --- | --- | --- | --- | --- | --- | --- | --- |
| **2** | 68 | 57.12 | **44** | 58 | 75.72 | **82** | 76 | 68.99 |
| **3** | 56 | 59.65 | **45** | 46 | 83.46 | **83** | 119 | 79.72 |
| **4** | 52 | 36.91 | **46** | 34 | 66.49 | **89** | 104 | 98.72 |
| **5** | 69 | 60.99 | **47** | 60 | 75.92 | **92** | 15 | 45.19 |
| **6** | 52 | 45.19 | **48** | 30 | 65.75 | **94** | 150 | 85.49 |
| **8** | 116 | 57.03 | **49** | 94 | 72.01 | **95** | 111 | 63.17 |
| **9** | 121 | 49.19 | **50** | 124 | 89.99 | **96** | 134 | 61.78 |
| **10** | 83 | 44.78 | **51** | 10 | 53.23 | **97** | 97 | 55.44 |
| **11** | 140 | 62.29 | **52** | 14 | 63.07 | **99** | 7 | 67.54 |
| **12** | 53 | 44.41 | **53** | 48 | 81.82 | **101** | 176 | 57.49 |
| **13** | 99 | 71.76 | **54** | 119 | 96.28 | **102** | 84 | 43.67 |
| **16** | 50 | 60.42 | **55** | 52 | 41.8 | **105** | 73 | 57.51 |
| **17** | 127 | 84.83 | **56** | 78 | 99.02 | **106** | 48 | 74.25 |
| **18** | 72 | 53.25 | **58** | 65 | 77.45 | **108** | 22 | 45.38 |
| **19** | 127 | 118.92 | **59** | 89 | 85.26 | **109** | 81 | 72.31 |
| **24** | 90 | 47.12 | **60** | 82 | 72.29 | **111** | 39 | 69.54 |
| **27** | 110 | 57.27 | **61** | 86 | 101.85 | **113** | 32 | 46.15 |
| **28** | 91 | 52.86 | **63** | 14 | 72.73 | **115** | 136 | 121.66 |
| **29** | 86 | 69 | **64** | 80 | 99.79 | **116** | 10 | 47.35 |
| **31** | 60 | 39.85 | **66** | 63 | 60.26 | **117** | 129 | 73.79 |
| **32** | 62 | 100.63 | **67** | 66 | 103.19 | **118** | 28 | 56.64 |
| **33** | 126 | 138.99 | **70** | 81 | 105.85 | **119** | 54 | 67.53 |
| **34** | 90 | 70.94 | **71** | 66 | 57.95 | **120** | 106 | 121.82 |
| **35** | 37 | 49.99 | **72** | 45 | 65.71 | **121** | 38 | 87.16 |
| **37** | 56 | 51.83 | **78** | 146 | 87.7 | **126** | 19 | 79.11 |
| **38** | 146 | 139.08 | **79** | 84 | 94.59 | **131** | 84 | 216.58 |
| **41** | 25 | 92.07 | **80** | 57 | 73.99 |  |  |  |
| **132** | 104 | 168.05 | **81** | 40 | 65.45 |  |  |  |

B: A brief description of questions with corresponding IDs as used in Fig 3a

| **Question ID** | **Question** | **Question ID** | **Question** |
| --- | --- | --- | --- |
| Q1 | Susceptible to illness | Q25 | Dry mouth |
| Q2 | Constipation | Q26 | Feelings of inadequacy |
| Q3 | Lowered self-confidence | Q27 | Easily startled/jumpy |
| Q4 | Loss of appetite | Q28 | Increased appetite |
| Q5 | Excessive sweating (e.g. hands, face, arm, pits etc) | Q29 | Impaired co-ordination |
| Q6 | Listlessness– don’t feel like doing stuff | Q30 | Uncertainty |
| Q7 | Forget things | Q31 | Become frustrated quickly |
| Q8 | Absentminded | Q32 | Biting of fingernails |
| Q9 | Feeling irritated | Q33 | Reduced motivation |
| Q10 | Nauseous | Q34 | Restlessness |
| Q11 | Pessimistic | Q35 | Poor judgment |
| Q12 | Jealous/Envious | Q36 | Increased smoking |
| Q13 | Moody | Q37 | Feeling out of control |
| Q14 | Feelings of depression | Q38 | Confused thoughts |
| Q15 | Anxiety | Q39 | Increased time sleeping |
| Q16 | Indecisive | Q40 | Waking up tired |
| Q17 | Unnecessary/excessive checking of work | Q41 | Feeling overwhelmed by demands |
| Q18 | Struggle to overcome minor | Q42 | Daydreaming |
| Q19 | Suspicious | Q43 | Procrastination |
| Q20 | Impaired concentration | Q44 | Feeling panicky |
| Q21 | Struggle to loose/gain weight even when following a diet | Q45 | Reduced productivity |
| Q22 | Skin disorders | Q46 | Wasting time on irrelevant activities |
| Q23 | Don’t take initiative as you used to | Q47 | Cannot discuss my problems with others |
| Q24 | Nightmares |  |  |
