# Supplementary material for: Estimation of Psychological Stress in Humans: A Combination of Theory and Practice
Source: PLoS One. 2013 May 15;8(5):e63044. doi: 10.1371/journal.pone.0063044 (PMC3654918; doi:10.1371/journal.pone.0063044)
Supplement: Table S4 — Metabolite IDs and references. (DOC) [file pone.0063044.s005.doc]

**Table S4**

A. Summary of metabolite IDs corresponding to ppm values and brief descriptions.

| **ID** | **ppm** | **Metabolite** |
| --- | --- | --- |
| Met5 | 4.3 | UK5 |
| Met10 | 4.13 | L-Dihydroorotic acid/Glyceric acid/3-Hydroxybutyric acid/  L-Lactic acid/(R)-3-Hydroxybutyric acid |
| Met12 | 4.11 | L-Dihydroorotic acid/L-Lactic acid |
| Met13 | 4.09 | Canavanine/6-Phosphogluconic acid |
| Met15 | 4.07 | Canavanine |
| Met16 | 4.06 | cyanocobalamin/Threonic acid/Canavanine/Hydroxyisocaproic acid |
| Met23 | 3.99 | isomaltose/Threonic acid |
| Met25 | 3.97 | isomaltose/Threonic acid/D-Cysteine/6-Phosphogluconic acid/L-cystiene |
| Met27 | 3.95 | aminoadipic acid/isomaltose/D-Arabitol/6-Phosphogluconic acid |
| Met30 | 3.92 | UK6 |
| Met31 | 3.91 | cynocobalamin/perillyl alchohol/D-Fructose |
| Met52 | 3.74 | cynocobalamin/aminoadipic acid/isomaltose/D-Arabitol/  N-Methyl-D-aspartic acid/Glutarylglycine/D-Tagatose/  3-Deoxyarabinohexonic acid/Gulonic acid/L-Iditol/Glyceric acid/Sorbitol/  L-Norleucine/Mannitol |
| Met53 | 3.73 | cynocobalamin/aminoadipic acid.isomaltose/  N-Methyl-D-aspartic acid/D-Xylitol/D-Tagatose/Gulonic acid/  L-Iditol/Glyceric acid/L-Alpha-aminobutyric acid/D-Fructose/L-Norleucine/Mannitol |
| Met54 | 3.72 | aminoadipic acid/isomaltose/D-Threitol/L-Iditol/Glyceric acid/Sorbitol/  L-Alpha-aminobutyric acid/D-Fructose/Mannitol |
| Met55 | 3.71 | isomaltose/D-Xylitol/Threonic acid/D-Tagatose/D-Threitol/Gulonic acid/  L-Iditol/L-Alpha-aminobutyric acid/D-Fructose/Mannitol |
| Met56 | 3.71 | isomaltose/D-Xylitol/Threonic acid/D-Tagatose/D-Threitol/Gulonic acid/  L-Iditol/L-Alpha-aminobutyric acid/D-Fructose/Mannitol |
| Met57 | 3.69 | Deoxycholic acid glycine conjugate/isomaltose/D-Arabitol/Threonic acid/  cysteine/D-Threitol/Gulonic acid/D-Fructose |
| Met58 | 3.68 | Deoxycholic acid glycine conjugate/isomaltose/D-Arabitol/Threonic acid/  cysteine/D-Threitol/Gulonic acid/D-Fructose |
| Met59 | 3.68 | Deoxycholic acid glycine conjugate/isomaltose/D-Arabitol/Threonic acid/  cysteine/D-Threitol/Gulonic acid/D-Fructose |
| Met60 | 3.67 | D-Arabitol/cysteine/D-Threitol/Gulonic acid/Sorbitol/D-Fructose |
| Met87 | 3.41 | cynocobalamin |
| Met88 | 3.4 | cynocobalamin/isomaltose |
| Met89 | 3.39 | 3,7-Dimethyluric acid/1,3,7-Trimethyluric acid |
| Met105 | 3.11 | D-Cysteine/Malonic acid//L-cystiene |
| Met147 | 2.15 | perillyl alchohol/D-Glutamine/L-Methionine/Sebacic acid |
| Met160 | 1.77 | aminoadipic acid/chenodeoxycholic acid/corticosterone/Glycocholic acid/  3-Deoxyarabinohexonic acid/Hydroxyisocaproic acid |
| Met161 | 1.62 | delta hexanolactone/aminoadipic acid/chenodeoxycholic acid/2-Methylglutaric acid/  3-Methyladipic acid |
| Met166 | 1.41 | delta hexanolactone/chenodeoxycholic acid/corticosterone/Glycocholic acid |
| Met169 | 1.2 | 3-Hydroxybutyric acid |
| Met170 | 1.14 | delta hexanolactone/chenodeoxycholic acid/corticosterone/Glycocholic acid |
| Met175 | 1.02 | Deoxycholic acid glycine conjugate/L-valine |
| Met177 | 0.99 | cynocobalamin/Deoxycholic acid glycine conjugate/Glycocholic acid/  Ethyl isopropyl ketone/Beta-Leucine/L-Alpha-aminobutyric acid |
| Met178 | 0.95 | cynocobalamin |

B. Reference table for metabolites corresponding to respective IDs as used in Figure 3d

| **Metabolite** | **Reports in literature** | **References** |
| --- | --- | --- |
| 3-hydroxybutyric acid | Elevated in Chronic and acute stress, Inflammatory bowel disease | [1,2] |
| 3-methyladipic acid | Elevated in ketosis | [3] |
| Aminoadipic acid | Elevated in Diabetes, renal failure and sepsis associated aging in tissue | [4] |
| Beta-leucine | Elevated in Liver cirrhosis and Acute liver disease | [1,5] |
| Chenodeoxycholic acid | Increased in Liver associated diseases | [6] |
| Cyanocobalamin | Decreased levels associated with depression  in AIDS patients,  Elevated levels associated with hepatic damage | [7,8] |
| D-Cysteine | Increased in Cardiovascular disease | [9] |
| Deoxycholic acid glycine conjugate | Increased in NAFLD | [10] |
| D-Threitol | Elevated in Hepatic cancer | [11] |
| Fructose | Increased levels associated with diabetes, hypertension | [12,13] |
| Glutarylglycine | Elevated in Mitochondrial energy  metabolism defects | [14] |
| Lactic acid | Increased in Hepatic cancer | [11] |
| L-valine | Increased in Hepatic cancer | [5,15] |
| Malonic acid | Elevated in Uremic patients, Esophaegal,  gastric, colorectal cancer | [3,16] |
| Mannitol/L-alpha-aminobutyric acid | Elevated in Acute renal failure | [17,18] |
| Sebacic acid | Increased level indicate defects in beta oxidation | [19,20] |
| Sorbitol | Increase associated with Hepatic cancer, Metabolic syndrome and associated  retinopathy | [11,21] |
| Threonic acid | Down-regulated in colorectal cancer | [22] |

**References:**

1. Teague CR, Dhabhar FS, Barton RH, Beckwith-Hall B, Powell J, Cobain M, Singer B, McEwen BS, Lindon JC, Nicholson JK*, et al.* (2007) Metabonomic studies on the physiological effects of acute and chronic psychological stress in Sprague-Dawley rats *Journal of Proteome Research* **6,** 2080-2093.

2. Shiomi Y, Nishiumi S, Ooi M, Hatano N, Shinohara M, Yoshie T, Kondo Y, Furumatsu K, Shiomi H, Kutsumi H*, et al.* (2011) GCMS-based Metabolomic Study in Mice with Colitis Induced by Dextran Sulfate Sodium *Inflammatory Bowel Diseases* **17,** 2261-2274.

3. Rhee EP, Souza A, Farrell L, Pollak MR, Lewis GD, Steele DJR, Thadhani R, Clish CB, Greka A, & Gerszten RE (2010) Metabolite Profiling Identifies Markers of Uremia *Journal of the American Society of Nephrology* **21,** 1041-1051.

4. Sell DR, Strauch CM, Shen W, & Monnier VM (2007) 2-Aminoadipic acid is a marker of protein carbonyl oxidation in the aging human skin: effects of diabetes, renal failure and sepsis *Biochemical Journal* **404,** 269-277.

5. Morgan MY, Milsom JP, & Sherlock S (1978) PLASMA RATIO OF VALINE, LEUCINE AND ISOLEUCINE TO PHENYLALANINE AND TYROSINE IN LIVER-DISEASE *Gut* **19,** 1068-1073.

6. Ferraris R, Fiorentini MT, Galatola G, Rolfo P, & Delapierre M (1987) DIAGNOSTIC-VALUE OF SERUM IMMUNOREACTIVE CONJUGATED CHOLIC OR CHENODEOXYCHOLIC ACIDS IN DETECTING HEPATOBILIARY DISEASES - COMPARISON WITH LEVELS OF 3-ALPHA-HYDROXY BILE-ACIDS DETERMINED ENZYMATICALLY AND WITH ROUTINE LIVER TESTS *Digestive Diseases and Sciences* **32,** 817-823.

7. Baldewicz TT, Goodkin K, Blaney NT, Shor-Posner G, Kumar M, Wilkie FL, Baum MK, & Eisdorfer C (2000) Cobalamin level is related to self-reported and clinically rated mood and to syndromal depression in bereaved HIV-1(+) and HIV-1(-) homosexual men *Journal of Psychosomatic Research* **48,** 177-185.

8. Rachmilewitz M, Stein Y, Aronovitch, & Grossowicz N (1959) SERUM CYANOCOBALAMIN (VITAMIN-B12) AS AN INDEX OF HEPATIC DAMAGE IN CHRONIC CONGESTIVE HEART FAILURE *Archives of Internal Medicine* **104,** 406-410.

9. El-Khairy L, Ueland PM, Refsum H, Graham IM, & Vollset SE (2001) Plasma total cysteine as a risk factor for vascular disease - The European Concerted Action project *Circulation* **103,** 2544-2549.

10. Barr J, Vazquez-Chantada M, Alonso C, Perez-Cormenzana M, Mayo R, Galan A, Caballeria J, Martin-Duce A, Tran A, Wagner C*, et al.* (2010) Liquid Chromatography-Mass Spectrometry-Based Parallel Metabolic Profiling of Human and Mouse Model Serum Reveals Putative Biomarkers Associated with the Progression of Nonalcoholic Fatty Liver Disease *Journal of Proteome Research* **9,** 4501-4512.

11. Li Z-F, Wang J, Huang C, Zhang S, Yang J, Jiang A, Zhou R, & Pan D (2010) Gas chromatography/time-of-flight mass spectrometry-based metabonomics of hepatocarcinoma in rats with lung metastasis: elucidation of the metabolic characteristics of hepatocarcinoma at formation and metastasis *Rapid Communications in Mass Spectrometry* **24,** 2765-2775.

12. Jalal DI, Smits G, Johnson RJ, & Chonchol M (2010) Increased Fructose Associates with Elevated Blood Pressure *Journal of the American Society of Nephrology* **21,** 1543-1549.

13. Kawasaki T, Akanuma H, & Yamanouchi TY (2002) Increased fructose concentrations in blood and urine in patients with diabetes *Diabetes Care* **25,** 353-357.

14. Bonafe L, Troxler H, Kuster T, Heizmann CW, Chamoles NA, Burlina AB, & Blau N (2000) Evaluation of urinary acylglycines by electrospray tandem mass spectrometry in mitochondrial energy metabolism defects and organic acidurias *Molecular Genetics and Metabolism* **69,** 302-311.

15. Xue R, Dong L, Wu H, Liu T, Wang J, & Shen X (2009) Gas chromatography/mass spectrometry screening of serum metabolomic biomarkers in hepatitis B virus infected cirrhosis patients *Clinical Chemistry and Laboratory Medicine* **47,** 305-310.

16. Nishiumi S, Shinohara M, Ikeda A, Yoshie T, Hatano N, Kakuyama S, Mizuno S, Sanuki T, Kutsumi H, Fukusaki E*, et al.* (2010) Serum metabolomics as a novel diagnostic approach for pancreatic cancer *Metabolomics* **6,** 518-528.

17. Thondorf I, Voigt V, Schafer S, Gebauer S, Zebisch K, Laug L, & Brandsch M (2012) Three-dimensional quantitative structure-activity relationship analyses of substrates of the human proton-coupled amino acid transporter 1 (hPAT1) *Bioorg Med Chem* **19,** 6409-6418.

18. Visweswaran P, Massin EK, & Dubose TD (1997) Mannitol-induced acute renal failure *Journal of the American Society of Nephrology* **8,** 1028-1033.

19. Rhee EP, Souza A, Farrell L, Pollak MR, Lewis GD, Steele DJ, Thadhani R, Clish CB, Greka A, & Gerszten RE Metabolite profiling identifies markers of uremia *J Am Soc Nephrol* **21,** 1041-1051.

20. Sim KG, Carpenter K, Hammond J, Christodoulou J, & Wilcken B (2002) Acylcarnitine profiles in fibroblasts from patients with respiratory chain defects can resemble those from patients with mitochondrial fatty acid beta-oxidation disorders *Metabolism* **51,** 366-371.

21. Dagher Z, Park YS, Asnaghi V, Hoehn T, Gerhardinger C, & Lorenzi M (2004) Studies of rat and human retinas predict a role for the polyol pathway in human diabetic retinopathy *Diabetes* **53,** 2404-2411.

22. Qiu Y, Cai G, Su M, Chen T, Zheng X, Xu Y, Ni Y, Zhao A, Xu LX, Cai S*, et al.* (2009) Serum Metabolite Profiling of Human Colorectal Cancer Using GC-TOFMS and UPLC-QTOFMS *Journal of Proteome Research* **8,** 4844-4850.
