# Supplementary material for: Estimation of Psychological Stress in Humans: A Combination of Theory and Practice
Source: PLoS One. 2013 May 15;8(5):e63044. doi: 10.1371/journal.pone.0063044 (PMC3654918; doi:10.1371/journal.pone.0063044)
Supplement: Table S5 — Significant metabolites and proteins. (DOC) [file pone.0063044.s006.doc]

**Table S5:** List of metabolites and proteins that showed significant differences among three groups (S, NS and BL) and their indirect and direct association with stress and different diseased states.

|  | **Direct association with PS** | | **References** |
| --- | --- | --- | --- |
| Metabolites | L-alpha-aminobutyric acid | | [1] |
| Proteins | HPT, TTHY, ALBU, IGHA2 | | [2,3,4,5,6] |
|  | **Indirect Association with stress** | |  |
|  | Stress Associated Diseases | Markers |  |
| Metabolites | Hepatic disorders | L-alpha aminobutyric acid | [1] |
| Proteins |  | HPT, TTHY, TALDO | [7] |
| Metabolites | Coronary disorders | Threonic acid | [8] |
| Proteins |  | APOA1 | [9,10] |
| Metabolites | Metabolic disorders | D-fructose | [11,12] |
| Proteins |  | ALBU | [13] |

## References

1. Fu JH, Sun HS, Wang Y, Zheng WQ, Shi ZY, et al. The effects of a fat- and sugar-enriched diet and chronic stress on nonalcoholic fatty liver disease in male Wistar rats. Dig Dis Sci 55: 2227-2236.

(a)

Figure 2

2. Joo Y, Choi KM, Lee YH, Kim G, Lee DH, et al. (2009) Chronic immobilization stress induces anxiety- and depression-like behaviors and decreases transthyretin in the mouse cortex. Neurosci Lett 461: 121-125.

3. Kohda K, Jinde S, Iwamoto K, Bundo M, Kato N, et al. (2006) Maternal separation stress drastically decreases expression of transthyretin in the brains of adult rat offspring. Int J Neuropsychopharmacol 9: 201-208.

4. Matos-Gomes N, Katsurayama M, Makimoto FH, Santana LL, Paredes-Garcia E, et al. Psychological stress and its influence on salivary flow rate, total protein concentration and IgA, IgG and IgM titers. Neuroimmunomodulation 17: 396-404.

5. Williams R, Speyer BE, Billing BH (1961) Serum haptoglobin in liver disease. Gut 2: 297-303.

6. Singh A, Smoak BL, Patterson KY, LeMay LG, Veillon C, et al. (1991) Biochemical indices of selected trace minerals in men: effect of stress. Am J Clin Nutr 53: 126-131.

7. Verhoeven NM, Jakobs C (2001) Human metabolism of phytanic acid and pristanic acid. Prog Lipid Res 40: 453-466.

8. Qiu Y, Cai G, Su M, Chen T, Zheng X, et al. (2009) Serum Metabolite Profiling of Human Colorectal Cancer Using GC-TOFMS and UPLC-QTOFMS. Journal of Proteome Research 8: 4844-4850.

9. Boisvert WA, Black AS, Curtiss LK (1999) ApoA1 reduces free cholesterol accumulation in atherosclerotic lesions of ApoE-deficient mice transplanted with ApoE-expressing macrophages. Arterioscler Thromb Vasc Biol 19: 525-530.

10. Aich P, Jalal S, Czuba C, Schatte G, Herzog K, et al. (2007) Comparative approaches to the investigation of responses to stress and viral infection in cattle. Omics: a journal of integrative biology 11: 413-434.

11. Jalal DI, Smits G, Johnson RJ, Chonchol M (2010) Increased Fructose Associates with Elevated Blood Pressure. Journal of the American Society of Nephrology 21: 1543-1549.

12. Depke M, Fusch G, Domanska G, Geffers R, Volker U, et al. (2008) Hypermetabolic syndrome as a consequence of repeated psychological stress in mice. Endocrinology 149: 2714-2723.

13. Abe T, Shono M, Kodama T, Kita Y, Fukagawa M, et al. (2004) Extracorporeal albumin dialysis. Ther Apher Dial 8: 217-222.
