# Supplementary figures and images for: Estimation of Psychological Stress in Humans: A Combination of Theory and Practice
Source: PLoS One. 2013 May 15;8(5):e63044. doi: 10.1371/journal.pone.0063044 (PMC3654918; doi:10.1371/journal.pone.0063044)

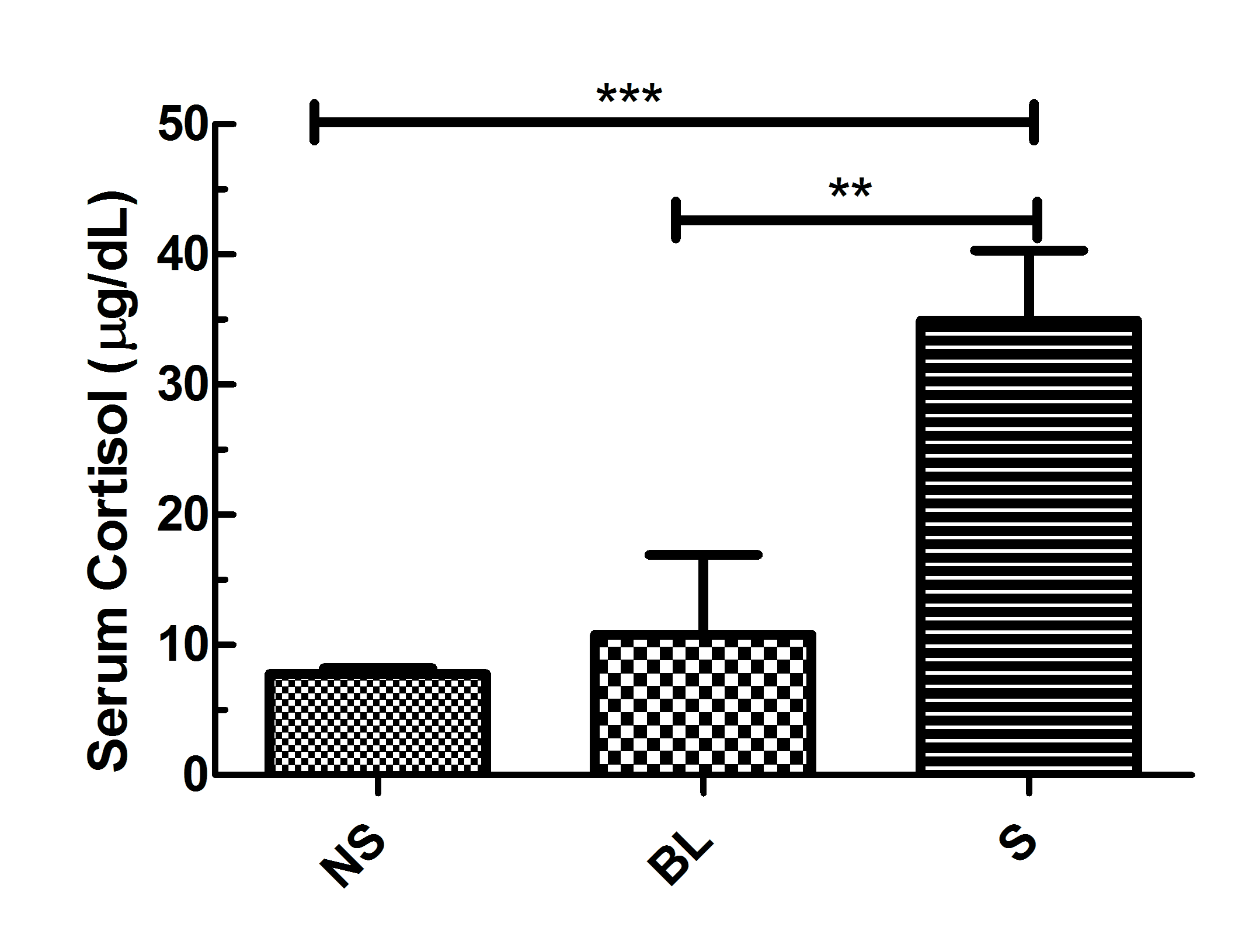

Supplement: Figure S1 — Absolute cortisol levels for three groups (BL, NS and S) are shown. Error bars shown are ±1SD of the data. Statistical significance between groups is also shown. (TIF) [file pone.0063044.s007.tif]
